# Spaying Bitches

**Published:** 1885-07

**Authors:** 


### EDITORIAL DEPARTMENT.

### SPAYING BITCHES.

Of this useful operation a certain English veterinary author* says: "Spaying, or removal of the ovaries of the bitch, is now almost unheard of, and I trust the time is not far distant when it will be discontinued on other animals. The operation is both inhuman and useless; I am thankful to say that I have never seen it performed in canine practice, and will, therefore, quote from another authority, Youatt." Hill then quotes from Youatt, who recommended that senseless operation by the flanks.

The only way to perform this operation is with ether, and by the median line. There is absolutely no danger in it; of 400 bitches of all ages, I have never lost one from the operation, and only one from the careless application of chloroform by an assistant, which caused me to have recourse to ether.

The operation is human and useful in that if made obligatory by law, except to breeders, in a special license, it will tend to decrease the number of dogs, especially useless curs, and thus lead also to a diminution of the chances of hydrophobia in man. There is a continual complaint among the farmers of the Eastern States "that they cannot raise sheep because of the damages done by dogs." Now the question is, What kind of dogs? cur dogs, that are raised at every hut and every cluster of houses at cross-roads through the country. Such people keep a dog, for what purpose nobody knows. A bitch is as good as any other, for to them a dog is a dog regardless of sex. It comes in heat; all the dogs of the neighborhood cluster round, snapping, snarling, and biting, and if one be rabid, may lead to the injury not only of people but of domestic animals.

If the law demanded that all bitches should be spayed, and that the operation should be certified to in regular form by the

* Woodruff Hill, "The Management and Diseases of the Dog."

operator, who should be a veterinarian, and then place the license of such spayed bitches at the same price as male dogs, and for unspayed bitches demand a license of $25—a breeder's license—the effect would be to lessen the number of curs in the country and increase that of valuable dogs which owners would look after. It may be said that this is unjust to the poor man, but it is not. If he wants a bitch he would then keep a good one, the young of which would in any year more than pay for the license.

Hill again says: "Breeding is a necessary process, and females prevented from it are sure to be affected with disease sooner or later."

That is downright false, and betrays an ignorance of the true conditions, only to be compared with the rest of his book on dogs. A spayed bitch is not to be compared to a castrated male, in any sense of the word. A spayed bitch is simply a natural one for ten months and a half of the year at least. She is just the same as when not in heat. She does not lie round, or accumulate fat, as the male dog does. She is the only decent house dog, and is absolutely free from the disgusting exposures and habits of the male dog. A spayed bitch is the only decent dog for a ladies' companion. A spayed bitch does not attract the filthy attentions of male dogs, even as much as other males, nor are they continually disgusting one by polluting every lamp-post and house corner, when one goes out for a quiet walk. They are not so liable to obesity and disease as other dogs, all authorities to the contrary notwithstanding.

Every government that has any interest in the welfare of humanity or agriculture, should compel the enforcement of these regulations.

